# Urinothorax—An Underdiagnosed Cause of Acute Dyspnea: Report of a Bilateral and of an Ipsilateral Urinothorax Case

**DOI:** 10.1155/2012/395653

**Published:** 2012-10-16

**Authors:** Leonidas Laskaridis, Spyridon Kampantais, Chrysovalantis Toutziaris, Basileios Chachopoulos, Ioannis Perdikis, Anastasios Tahmatzopoulos, Georgios Dimitriadis

**Affiliations:** Urologic Department, Aristotle University of Thessaloniki, 54635 Thessaloniki, Greece

## Abstract

Urinothorax (UT) is a rare and often undiagnosed condition, defined as the presence of urine in the pleural cavity due to the retroperitoneal leakage of urine accumulation, known as urinoma, into the pleural space. UT usually is a transudative pleural effusion that presents in patients with obstructive uropathy and it may occur following surgical procedures in the ureter or kidney such as ESWL, PCNL, and URS. Its diagnosis requires a high degree of clinical suspicion since the respiratory symptoms tend to be absent or mild and the urological signs tend to dominate. However, UT may rarely present with severe and acute dyspnea as well. The objectives of this study are to describe two new cases of this rare entity, a bilateral case and an ipsilateral case focusing on the side that occurs according to the affected renal insult, and to alert the physicians to include UT in their differential diagnosis of pleural effusions especially in patients with recent urinary tract disorders.

## 1. Introduction

The presence of urine inside the pleural space is called urinothorax. It is a rare cause of pleural effusion secondary to (i) traumatic or (ii) obstructive causes [1]. Initially it was described by Corriere et al. in 1968 in their studies of ureteral obstruction in dogs [2]. Nowadays, cases of urinothorax have been described in human patients. At the present time, increased awareness of this entity coupled with the availability of advanced imaging and scintigraphic techniques have resulted in the increase of cases of urinothorax being diagnosed [3].

## 2. Case 1

**Figure 1:**
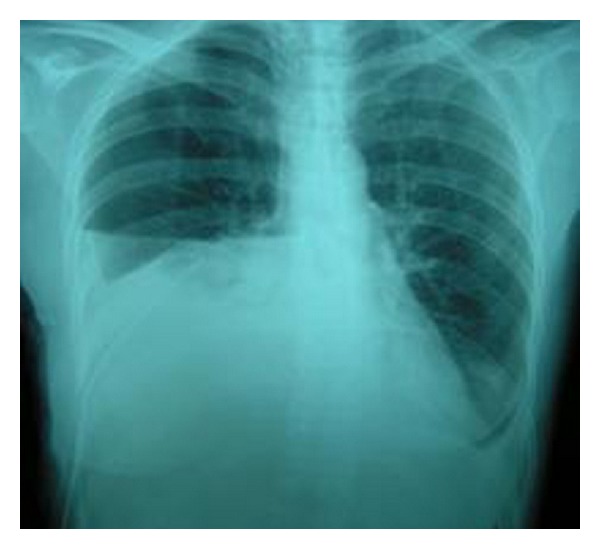
Case 1: chest radiograph showing pleural effusion on the right side.

**Figure 2:**
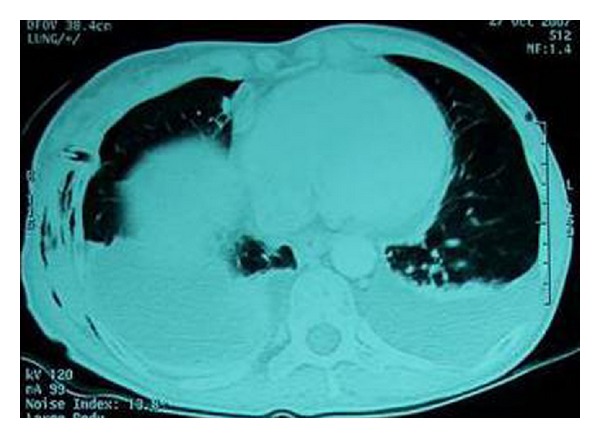
Case 1: CT scan revealing extensive bilateral pleural effusion.

A 45-year-old woman was referred to our department with complaints of high-grade fever and progressive shortness of breath. The patient had undergone diagnostic right ureteroscopy for recurrent renal colic at a different centre one week ago. She had a history of gastrectomy due to gastric carcinoma three years earlier, with subsequent chemotherapy and irradiation. There was no past history of pulmonary or heart disease. On clinical examination, the patient was febrile with a high respiratory rate. Chest radiograph showed massive pleural effusion on the right side (Figure 1) and chest CT (computed tomography) confirmed the diagnosis revealing bilateral pleural effusion (Figure 2). The patient was emergently managed with right closed pleural drainage. Biochemical evaluation of the drainage fluid indicated that it was urine. IVU (intravenous urography) and abdominal CT demonstrated retroperitoneal urine accumulation. This was a possible iatrogenic complication during the previous ureteroscopy. After the subsiding of the respiratory symptoms this urine collection was further treated with percutaneous nephrostomy and drainage of the retroperitoneal space. The chest tube was removed on the fourth day without recurrent effusion.

## 3. Case 2

**Figure 3:**
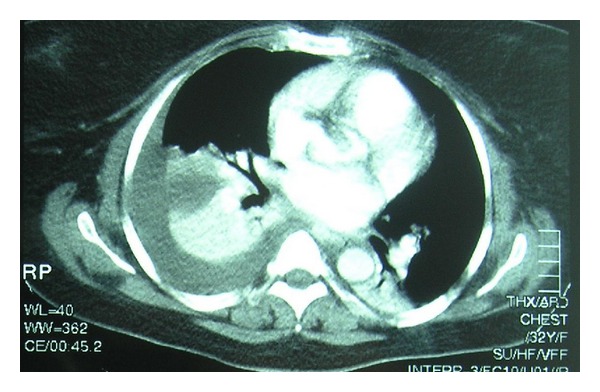
Case 2: CT scan revealing ipsilateral pleural effusion.

**Figure 4:**
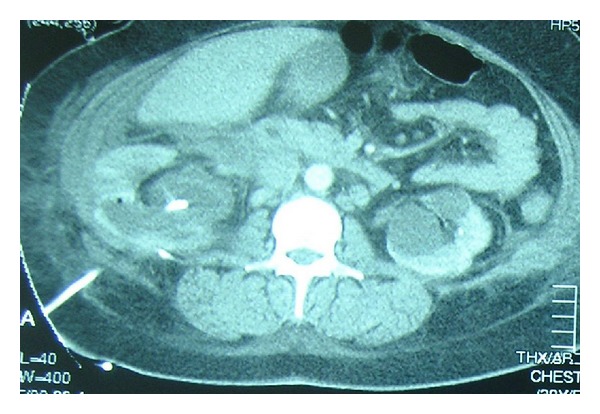
Case 2: CT scan showing the placement of nephrostomy tube.

A 32-year-old woman was presented to our department with symptoms of right renal colic. One year earlier, after the birth of her second child, the woman had undergone anatrophic nephrolithotomy in our hospital for the management of a large staghorn calculus. On initial presentation to the emergency room, the patient had right flank pain consistent with ureteral obstruction. Her admission chest X-ray film was normal. The KUB (kidney, ureter, and bladder) film showed a stone in the lower third of right ureter and the US (ultrasound) revealed associated hydronephrosis. Because of her past surgical history, her admission was decided in order to perform ureteroscopic lithotripsy. Next day and before the planned endoscopic management, the patient began to experience right pleuritic pain and sudden severe dyspnea developed. Her admission to the ICU (intensive care unit) was mandatory. A pulmonary embolism was the initial possible diagnosis. Chest CT revealed a right pleural effusion and perihepatic fluid collection in the more caudal images (Figure 3). Therefore scanning of the entire abdomen was performed which demonstrated a large hydronephrotic kidney with concomitant urine extravasation. A right chest tube and a nephrostomy tube were placed for drainage (Figure 4). The pleural fluid revealed a creatinine level higher than serum which demonstrated that consisted of urine. The patient had a long course in the ICU with persistent fever and an episode of reversible acute renal failure. After 20 days, a repeat X-ray showed an expanded right lung with no residual pleural effusion even after clamping the intercostal tube drainage. This drain was therefore removed and the patient discharged with the nephrostomy tube that remained for approximately one year when surgical removal of the obstructive stone was performed.

## 4. Discussion

Urinoma is the accumulation of urine outside of the urinary tract in the retroperitoneal space by a leakage originating from the kidney or an injured ureter [4]. Usual causes of urinoma's formation are complications of surgical procedures in the kidney or the ureter (perforation, PCNL, ESWL), retroperitoneal inflammation, trauma, urinary obstruction, and malignant diseases [5–8]. Some of the accumulated urine may pool in the pleural space, forming a pleural effusion. There are two theories under debate, concerning the mechanisms responsible for this transdiaphragmatic evasion of urine: (i) urine may travel through lymphatic drainage into pleural space or (ii) retroperitoneal urine firstly enters the peritoneal cavity and afterwards travels through lymphatic drainage into the pleural cavity—however, there is no evidence supporting this theory in human patients [7, 9].

Often, we are presented with patients suffering from urinothorax complaining of dyspnea, flank pain, and fever and who have on clinical examination high respiratory rate [4, 10]. For the detection of UT, multiple radiographic examinations are available. The most common one of them, the chest radiograph, always shows extensive pleural effusion. Intravenous pyelography (IVP) can reveal the leakage of contrast from the retroperitoneal space to pleural cavity, but sometimes this examination may prove to be noncontributary [8, 11]. Abdominal and thoracic CT scanning is indispensable in the detection of pleural effusions and of underlying urinoma. Renal scintigraphy with the use of technetium-99m DTPA, technetium-99m ethylene dicysteine (EC), or with technetium-99m-mercaptoacetyltriglycine-3 can also be utlized to reveal any extravasation of urine from the region of the kidney or the ureter [8, 9, 11]. The diagnosis of urinothorax is accomplished through thoracocentesis by acquiring fluid from the pleural effusion in order to examine it biochemically [3]. The fluid is usually straw colored with the distinctive smell of urine [1]. In the majority of the cases, it is revealed to be a transudate according to Light's criteria, with biochemical test characteristics of low glucose, low Ph, a possibly elevated LDH level, and low protein concentration [1, 10, 12]. The most important biochemical parameter though is the fluid creatinine-to-serum creatinine ratio which is higher than 1 and in most cases greater than 10 [1, 3, 10, 13, 14].

The treatment of urinothorax is relatively straightforward. The correction of the underlying cause usually suffices, resulting in the spontaneous resolution of the urinothorax [5, 15]; if the pleural effusion persists, then drainage of the urine through an intercostal thoracic tube is recommended [3, 15, 16].

The majority of the cases of urinothorax are ipsilateral with the urinoma. However, there are rare cases of bilateral and contralateral urinothoraces [3, 9, 17]. In our cases, we were presented with a patient with a bilateral urinothorax (Case 1) and a patient with an ipsilateral urinothorax (Case 2).

In conclusion, although UT's occurrence is rare, physicians need to maintain a high degree of clinical alertness and include it in the differential diagnosis of pleural fluid effusions, especially in the case of patients with recent urinary tract disorders and surgical procedures. Its presentation is usually ipsilateral to the urinoma; however it may also present bilaterally or contralaterally. Correction of the underlying cause is usually sufficient for the spontaneous and prompt resolution of UT although a drainage tube can be utilized as well.
